# Pleurisy with Effusion

**Published:** 1872-07

**Authors:** Richard B. Maury

**Affiliations:** Professor of Theory and Practice of Medicine in Memphis Medical College


ATLANTA

### Medical and Surgical Journal.

Vol. X.—JULY, 1872.—No. 4.

---

### ORIGINAL COMMUNICATIONS.

---

### PLEURISY WITH EFFUSION.

BY RICHARD B. MAURY, M.D.,

*Professor of Theory and Practice of Medicine in Memphis Medical College.*

[Before the Clinical Society of Memphis, Tennessee.]

Mr. President,—For the purpose of bringing more forcibly before the Society the prominent features of pleurisy with effusion, I have thought it proper to present the following history:

L. S., a boy aged five years and eight months, was seized with violent fever on the evening of the 3d of February, 1872, after marked exposure to cold. I saw him, in connection with Dr. Williams (with whose permission the case is reported), on Monday evening, February 5th. The case was considered by us both to be one of fever, aggravated by intestinal accumulations of indigested apples, oranges and nuts. He had been passing these substances in large quantities before my visit, and his abdomen was still much swollen. The pulse was one hundred and sixty per minute, and the respiration fifty. There was extreme restlessness, with great nervous excitement. Marked epigastric fulness, with general abdominal distension, interfered with free action of the diaphragm.

Under active treatment, defervescence occurred on the night of the 8th of February. About eight A.M. on 9th February, slight rigors, with cool extremities, introduced a pneumonia at the left base. The fever again rose, the respiration became hurried, and the characteristic red blush appeared on the left cheek. There was added, also, decided cough, with expectoration of viscid, tenacious and bloody sputa. Physical exploration revealed increased fremitus upon palpation; dulness on percussion, with moist rales; and subsequently, bronchial breathing and bronchophony on auscultation.

The pneumonia, with the usual medical aid, ran its course in eight days. The pulse came down to ninety-five, and the respiration to thirty per minute, and the boy's condition seemed very favorable, when, on the morning of the 18th February, he began to complain of pain in the left side, below and behind the nipple. The breathing again became hurried, and the pulse rose to one hundred and thirty per minute. These symptoms were regarded as indicative of an attack of pleurisy, and in the course of forty-eight hours, marked effusion into the left pleural sack, was demonstrable. The decubitus now was striking and characteristic, being forward and to the left or diseased side—so that the little patient could not lie, but had to be supported by pillows in a half-sitting, half-prone position.

The administration of calomel, squills and digitalis internally, the application to the chest of a blister, and afterward of tincture of iodine, failed to promote absorption of the effusion. Iodide of potassium, also, was used internally, but without benefit.

The patient's condition, for some time, underwent little or no change. The pulse stood regularly at from one hundred and forty to one hundred and forty-five per minute; the respiration forty-five to fifty per minute. The cough continued, but was not at all troublesome, and was accompanied with slight whitish expectoration. By-and-by, however, the face became puffy and swollen, and the superficial vessels over the cheeks became enlarged.

Matters stood thus, without material loss or gain for the patient, until the 15th of March. At this time, percussion resonance at the apex was very greatly impaired, while over the lower portions of the left lung, at and near its base anteriorly and posteriorly, percussion elicited absolutely flat sounds. The heart's apex struck the chest wall a little to the right of the sternum. The intercostal depressions over the diseased side were obliterated, and the left chest did not participate appreciably in the respiratory movements. Vocal fremitus was null over the same side; but bronchial breathing and voice were still distinct, even at the base where percussion dullness was most marked. The patient was very intractable, and no accurate measurement of the chest could be made; but to the eye, the left side evidently bulged more than the right.

We decided to resort to paracentesis on the 15th of March, the twenty-sixth day of the pleurisy, but the illness of the child's father, who was exceedingly anxious to be present at the operation, induced us to postpone it until the 19th of March. On this day, the patient being under chloroform, an exploring needle was first passed into the cavity of the chest at a point situated two inches above the lower limit of pulmonary resonance, posteriorly on the healthy side, and in a line dropped perpendicularly from the angle of the scapula. The vacuum syringe being now attached, the effusion, to our disappointment, was found to consist of thick, yellow, healthy-looking pus.

A trocar was then introduced just where the exploring needle had entered, and a Davidson's syringe being properly attached, twenty-eight ounces of thick, yellow pus were drawn off, without the admission to the chest of a single bubble of air. The canula being withdrawn, we concluded to close the wound with adhesive plaster, and await the course of events. The patient spent a comfortable night, and the next day seemed much refreshed, and more disposed to take food than at any time during his illness. The pulse was one hundred and twenty per minute, and respiration forty-five. Skin cool and pleasant.

March 24th.—Is not doing well. Pulse one hundred and forty-eight, and respiration sixty-two per minute. Has a good deal of cough, with free muco-purulent expectoration. Dullness upon percussion, and other physical signs nearly the same as before the operation. His appetite, however, has been much improved, and he begs for food every hour. By means of a bistoury, an opening was made at the point previously selected for paracentesis. The canula was then introduced, and the fluid allowed to drain away, no suction being employed. Somewhat less than one pint of thick, healthy-looking pus having escaped, a drainage arrangement, made of silver wire, was introduced, and secured in position by adhesive strips.

March 27th.—Pus is being discharged freely. The appetite is voracious. Pulse one hundred and twenty-four, and respiration thirty-six per minute. The cough very greatly lessened. The nurse is instructed to turn the drainage wire entirely around in the opening every day.

April 25th.—The patient has slowly but steadily improved. His appetite is extraordinary; he eats something every hour of the day, and occasionally has diarrhœa in consequence. There is slight febrile excitement in the evening, but he awakes refreshed in the morning. Has gained in weight seemingly, and certainly in strength. He spends the day sitting on the bed, or being wheeled about the house and in the yard in his carriage; is now beginning to use his legs in walking a little. The chest is decidedly flattened anteriorly on the diseased side, but the air is found to enter the lung generally and freely. It is difficult, if not impossible, to say precisely where the heart's apex strikes the chest wall. Upon auscultation, the first sound is heard most distinctly just beneath the lower third of the sternum. There is now no cough. The breathing, though abnormally frequent, is tranquil and regular. The pulse is from one hundred and twenty to one hundred and thirty per minute. The amount of pus discharged in twenty-four hours is less than a fluid-ounce. It is free from offensive odor, and no air seems to have entered the chest. The drainage wire has accomplished its object satisfactorily.

The prospect of recovery would seem to be very fair, but, as we shall see in the remarks that follow, the result is necessarily very uncertain.

It is scarcely necessary to remark, Mr. President, that the case here reported is not one of simple pleurisy with effusion. The pleuritic inflammation in our little patient was not the primary disorder, but was engrafted upon, or secondary to, the the febrile disease and the pneumonia which preceded it.

It is a well-known fact, that pleurisy may complicate, or be secondary to, many of the acute fevers. It may appear in the course of the exanthemata, or it may complicate typhoid fever, and then the prognosis is not nearly so favorable as in the simple or primary cases. The patient's strength has already been seriously undermined by the fever, and when the pleurisy supervenes, it is almost sure to pursue a chronic course, and to be attended by a purulent effusion.

Again: it is the rule for a dry pleurisy, or adhesive inflammation of the pleura, to appear as part and parcel of every pneumonia which involves the superficial portions of the lung; but it is comparatively rare for effusion into the pleural sack to accompany the pneumonia as it did in the case of our patient. When, however, it does so, the pleurisy is very unmanageable, and the prognosis grave.

When we come to inquire into the etiology of this disease, I must state that very little seems to be known in regard to it. There is a popular notion that it is due to cold, and this was for a long time a prevailing idea with our profession; but of late we find many of the most intelligent observers denying utterly the existence of such a cause.

In the case before us, the history states that the fever came on after marked exposure to cold; but then defervescence was complete on the fifth day, and the pleurisy did not make its appearance for eight or nine days after.

We find Anstie and Hyde Salter, Fuller and Ziemssen, and others denying the truth of the old notion that pleurisy is produced by exposure to cold. Ziemssen minutely examined the history of fifty-four cases in reference to this point, and could not ascribe the attack to cold in a single one.

I must confess my inability to understand how cold alone could produce inflammation of the serous membrane. We can easily enough conceive of the case of an individual whose blood, from excess in eating and drinking, is loaded with materials which can not be appropriated by the system, and who, by exposure to cold, has the functions of the kidneys and skin almost entirely suspended. In this case there would be a blood-poisoning, such as occurs from retained excreta under different circumstances—a condition which clinical observation has shown to be attended by local inflammations: *e. g.*, angina faucium, pleurisy, and inflammation of the joints, as recorded by Dr. Parkes. But here the exposure could, at most, be regarded only as an exciting cause—one which, at farthest, could only develop influences which, though preëxistent, were dormant.

Under the head of symptoms, I will advert to two only. The first is the quality of the pulse. Most authorities describe it as hard and concentrated, or hard and bounding. This supposed feature was regarded as a prominent indication for general bleeding in time past. In the present case, the pulse was not of this character; nor has it seemed to me full and hard in any of the few cases of acute pleurisy which have occurred under my observation of late years. But I alluded to this point especially to draw the attention of the Society to the result of Dr. Austie's observations with the sphygmograph; this, he says, "uniformly shows that the large and somewhat bounding pulse is always decidedly less resistent than that of health."

A marked feature of pleurisy, and one in which it differs materially from pneumonia, is the absence of any established thermometric range. While, in acute pneumonia, defervescence is apt to occur by crisis on the fifth, sixth or seventh day, in acute pleurisy, the temperature follows no regular course whatever.

I do not propose, now, to say anything upon the subject of the physical signs presented in pleurisy, any further than to invite attention to a peculiarity which is usually met with in the pleurisy of children. This peculiarity was very marked in the case before us, and the practitioner who is not aware of it may very easily be embarrassed by it. After we had selected what was deemed the proper place upon the surface of the child's chest for the introduction of the trocar, it was found that bronchial breathing and bronchophony were to be heard with the utmost distinctness in this very spot. Although the chest was nearly full of fluid, and the lung very much compressed, and although percussion dullness was marked, and vocal fremitus was null at the base of the chest, these two signs were still clear and distinct. This might have made us hesitate about tapping the chest at that point had not Rilliet and Barthez, and afterward Anstie, called attention, in a most emphatic manner to this peculiarity which is presented in the pleurisy of childhood; I mean the persistence of bronchial breathing and bronchial voice over the lower portions of the chest, even though the lung may be crowded into the upper part by excessive effusion. Any doubt, however, which might linger upon our mind will be certainly and safely removed by the introduction of the exploring needle.

Among the interesting sequelæ which may appear in the track of a pleurisy which has become chronic, I shall dwell upon one only, and that is pulmonary tuberculosis. When I was a young graduate, thirteen or fourteen years ago, the proposition was thus stated by the practical men of that time: If a pleurisy resists treatment, becomes chronic, and eventuates in purulent effusion, it does so, generally, because of the existence of a tuberculous diathesis; and the proof of this proposition was supposed to be furnished in the fact that such patients often die of phthisis. Now, however, I believe, we are justified in placing quite a different construction upon this fact.

Von Troltsch has remarked upon the great frequency with which the subjects of chronic purulent discharge from the ear die of tubercular meningitis.

Dr. Simpson, some years ago, without reference to this subject, commented upon the liability of persons with chronic pelvic abscess to die of tubercular peritonitis. All modern observers from Rokitansky to the present time, are agreed that the occurrence of acute tuberculosis is preceded, with the fewest exceptions, by the formation of old purulent or cheesy deposits somewhere in the body.

Some years ago I lost a patient from vesico-intestinal fistula, caused by an abscess situated between the bladder and sigmoid flexure, the mucous surface of the bladder was found to be studded with tubercles.

Lastly, the results recently obtained by the inoculation of tubercle and other organic products have such an unmistakable bearing upon this subject, that it is only necessary for me to allude to them here to be understood.

The relation existing between chronic purulent effusion into the pleura and the tuberculosis which follows would, therefore, seem to be properly explained thus: The detritus of inflammatory products, having obtained admission to the circulation, passes along with the blood until it reaches the smallest capillaries of the adjacent tissues; small particles, here finding lodgment, act as so many centers of irritation, and each one of these centers soon becomes occupied by a little clump or cluster of tubercle cells. Hence, in chronic accumulations of pus in the pleura, the tubercles will be found deposited upon the surface of the false membranes, upon the pleura itself, and in the adjacent portions of the pulmonary parenchyma.

The practical lesson learned from this knowledge of the pathology is to give early vent to collections of pus, and afford them free and uninterrupted drainage.

A few words now in regard to the pathological anatomy.

Inflammation of the pleura may result in an exudation of fibrinous matter, and nothing more, upon the surface of the serous membrane. Usually, however, there is, in addition to the fibrinous material, an abundance of serum poured into the serous cavity. This may be absorbed, or, after remaining serum for a long time, it may become converted into pus. Lastly, the effusion may be pure pus from the beginning.

In the pleurisy which comes on as part and parcel of acute pneumonia, or that which complicates consumption, the products of inflammation are, as a rule, fibrinous, and only fibrinous, and the disease eventuates in adhesion of the opposing serous surfaces. In acute and chronic primary pleurisies, we expect to find fibrinous products and serum together—the serum greatly predominating. In the pleurisy which complicates occasionally the acute fevers—in that which occurs in scarlatina—the effusion from the very start may be purulent. Indeed, I have thought it was purulent from the beginning in this case which I have reported—partly from the fact that the pleuritic inflammation was secondary to the fever, and partly because the chill and the hectic flush, which usually attend the change from a serous to a purulent fluid, were wanting. Dr. Williams and I carefully watched for any symptoms which might indicate such a change, but discovered none.

The knowledge that an effusion may be purulent from the beginning, suggests to us the importance of being prepared to resort to paracentesis at a very early period in the history of those pleurises which complicate or are secondary to the acute fevers. If any one desires to go more fully into the study of this portion of our subject, he will find the details of the formation of pus upon a serous surface most satisfactorily given and illustrated by Rindfleisch, in his "Pathological Histology."

An opportunity for studying the primary formation of pus upon serous surfaces is more often afforded in peritoneal inflammations than in pleuritic. In the examination of the peritoneum after death from puerperal fever, I have repeatedly seen the pelvic viscera covered with pus from an inflammation which had not existed more than forty-eight or seventy hours.

But perhaps the most interesting question to be answered in this case is, In what condition will the lung be left in the event of recovery?

It should be remembered that the once smooth serous surface of the pleura is now covered with layers of false membrane many lines in thickness. Into the substance of this membrane, new blood-vessels have forced their way, and upon its free surface, capillary plexuses ramify in great abundance. This free surface is covered with exuberant granulations, and from it, pus is freely poured into the pleural cavity. An artificial opening having been made, and free drainage established for the pus, the following result may be obtained: The lung does not resume its original volume, but expands to a greater or less extent, in proportion to the length of time it has been under pressure, and the strength of the false membranes which confine it to its new position. New granulations, rapidly increasing upon the whole suppurating surface, contribute to fill up the cavity left by the removal of pus, just as granulations fill up gaps in the tissues from surface wounds.

But the most potent agent at work to prevent the formation of a vacuum in the cavity of the chest is the true cicatricial tissue which is found in great abundance in the pseudo-membrane. The delicate chest walls of the child yield greatly under the pressure of the atmosphere, and become flattened; but being made of bone, a limit is soon reached, beyond which they can not yield. The influence of cicatricial tissue is most marked in burns, but it is very powerful here also. It confines and compresses the lung; it drags the diaphragm into the cavity; it draws together the opposing walls of the chest, as iron bars, cooling from a high temperature, approximate walls of masonry. Under its influence, also, the shoulder drops, and is drawn forward, and the anterior chest wall, no longer convex, becomes flat and even concave at its upper part. As Rindfleisch expresses it, the indurated sack into which the pleura has been converted, contracts as the urinary bladder contracts. The cavity is thus at last obliterated, and the fistulous opening closes. For months or years, the patient is more or less of an invalid; but usually the crippled lung recovers in great measure its functions, and no serious trouble is left, especially if the disease has occurred in early life.

In the treatment of acute pleurisy, our first object is to relieve pain and secure perfect rest, so that the inflammation may run its course under the most favorable circumstances. This object is to be accomplished by the use of morphia internally, and by the application to the chest of cups or leeches, with hot fomentations. It has seemed to me, in the treatment of pleurisy, that morphine should be combined with antimony and sulphate of magnesia, in minute doses, for the purpose of obviating its drying effect upon the secretions. We should do nothing which might hinder the removal of the effusion.

Our first object being accomplished, attention should then be turned to the effusion. The fever having abated, a large blister is to be applied over the diseased side, and an attempt made to remove the fluid from the pleura by the use of diuretics. If, however, the urine is scant, and the mouth and skin are dry, the old pill of calomel, squills and digitalis may be employed until its effect upon the secretions is perceived. It is then to be discontinued, and the iodide of potassium given in eight or ten grain doses, three times a day. The affected side should again be blistered as soon as possible. I have never seen any positive results from the external use of iodine, in weak or strong solutions, and prefer to repeat the blister.

Under such treatment, or even less, many cases of pleurisy with effusion will go on steadily toward recovery. Indeed, in some cases, the intervention of art is not required any further than it may be necessary to relieve pain and procure rest. In other cases, the effusion increases uninterruptedly, in spite of all treatment, until the patient is on the verge of suffocation.

There is one fact which should be remembered, and that is, the poverty of our therapeutic resources for promoting the absorption of effusions. Bearing this in mind, we should guard against doing too much, and after waiting a reasonable time, should, if medicine fails, resort to surgery for relief.

How long a time shall the physician wait in order to decide whether the fluid is going to be removed by medical means?

The rule adopted, and which I consider a safe one, is not to wait longer than three weeks in the case of a child, nor more than four in an adult, provided we are sure, at the end of such time, that the effusion is not diminishing. Paracentesis should then be resorted to. If the diminution is so slight that we are in doubt whether the fluid is really lessening, the safest plan still is to tap. If the effusion has collected rapidly, so as to seriously embarrass respiration, tapping may be resorted to at any period, however early, if it seems necessary for relief.

A few words now in regard to the operation itself. When performed with care and intelligence, it is entirely free from danger; and when divested of the complicated suction apparatus generally employed, it is as simple an operation as paracentesis of the abdomen. The steps of the operation have already been detailed in the history of our case. A common Davidson's syringe was used on this occasion; but even that might have been safely dispensed with—indeed, it was not used at the second operation.

In purulent effusions, no one uses a suction apparatus. The reason assigned for its use in serous effusions is to prevent the admission of air to the chest. In reference to this point, I will state that I have seen air admitted in several cases without any injury. Very recently, I tapped the chest of a lady, and using the syringe for withdrawing the fluid, a considerable amount of air was admitted; notwithstanding this, the result was eminently satisfactory. This is the experience of others, also.

Again: Why should air enter the pleura, if paracentesis be performed with a trocar and canula simply, any more readily than it does the peritoneum? Last week, I tapped a lady for the fifteenth time, for abdominal dropsy. The fluid was as clear then as at the first tapping, and I do not remember that air gained admission but on one occasion.

At the close of the operation, when the fluid is nearly exhausted, a fit of coughing, by exaggerating the inspiratory movement may draw air into the chest; but a repetition of this is easily prevented by placing the finger over the canula. I see no reason why we can not dispense with the suction apparatus altogether, taking care to close the opening carefully on the withdrawal of the canula. For obvious reasons, an effusion into the pleura can not be as completely removed as one into the cavity of the abdomen. The fluid will continue to flow as long as the lung is able to expand and the chest walls to yield; it will then stop. It is idle to talk about emptying it all, except in cases of very rapid effusion. Usually, the removal of a very moderate amount of the effusion is followed by the rapid absorption of the remainder.

After the operation, it is wise to stimulate and build up the system in every possible way. Some patients do well on tincture of bark and good diet. To others, it is better to give iron in the form of muriated tincture or ammonio-citrate; to others again, the iodide of iron and cod-liver oil.

NOTE.—At the present date—June 14th, 1872—this little patient is doing well; spends most of his time in the open air, and is preparing to go to Colorado, to remain till the Fall.

